# Three-Dimensional-Printed Thermoplastic Polyurethane (TPU) Graft and H-Button Stabilization System for Intra-Articular Cranial Cruciate Ligament Reconstruction: Cadaveric Study

**DOI:** 10.3390/vetsci12080725

**Published:** 2025-07-31

**Authors:** Menna Nahla, Yara Abouelela, Mohammed Amer, Marwa Ali, Abdelbary Prince, Ayman Tolba, Ayman Mostafa

**Affiliations:** 1 Department of Small Animal Surgery and Radiology, College of Veterinary Medicine, Cairo University, Giza 12211, Egypt; mnnatya@gmail.com (M.N.); mohammedvet@cu.edu.eg (M.A.); 2 Department of Anatomy and Embryology, College of Veterinary Medicine, Cairo University, Giza 12211, Egypt; yarasayed89@cu.edu.eg (Y.A.); draymantolba@hotmail.com (A.T.); 3 Department of Spinning and Weaving Engineering, Textile Research and Technology, Institute National Research Centre, Giza 12622, Egypt; marwaatf@gmail.com; 4 Department of Biochemistry and Molecular Biology, College of Veterinary Medicine, Cairo University, Giza 12211, Egypt; bioproteomics@cu.edu.eg; 5 Department of Veterinary Clinical Sciences, College of Veterinary Medicine, Western University of Health Sciences, Pomona, CA 91766, USA

**Keywords:** canine CrCL, stifle, cruciate, reconstruction, 3D-printing, TPU, polyurethane, graft

## Abstract

Cranial cruciate ligament (CrCL) rupture is a common orthopedic disorder in dogs, leading to stifle joint instability and progressive osteoarthritis. This study aimed to develop and biomechanically evaluate a novel intra-articular reconstruction system designed to mimic the natural ligament and restore joint stability following CrCL excision. The system consisted of a 3D-printed thermoplastic polyurethane (TPU) graft, cerclage wire, and H-button fixation. Fourteen pelvic limbs from mature mixed-breed cadaveric dogs were used. The inclination angle, dimensions, volume, tensile strength, and elongation of the native CrCL were measured. Seven CrCL-deficient stifles were reconstructed using the proposed system and tested biomechanically. The native CrCL showed a significantly higher tensile strength than the TPU graft; however, the TPU demonstrated a greater flexibility. The reconstruction system successfully stabilized the joint and provided repeatable fixation. Significant correlations were found between CrCL volume and both age and body weight. These findings support the mechanical suitability of the proposed system for ex vivo stifle stabilization and highlight the potential of 3D-printed TPU in ligament reconstruction. Further in vivo studies are recommended to assess long-term performance, including implant integration, tissue remodeling, and clinical outcomes.

## Simple Summary

Rupture of the cranial cruciate ligament is a major cause of knee pain and lameness in dogs. In this study, a completely new system was developed to reconstruct the damaged ligament using advanced 3D printing technology. The system included a flexible graft made from a material called thermoplastic polyurethane, along with a specially designed H-shaped button plate and a supportive wire to secure the implant in place. This design was tested on dog knee joints from cadavers to see how well it could restore joint stability after ligament excision. The results showed that the new system provided good mechanical support and successfully stabilized the joint. The flexible graft allowed for natural movement, while the other parts held everything securely. This study shows promise for improving surgical options to treat knee injuries in dogs, with the goal of restoring mobility, reducing pain, and enhancing the long-term well-being of affected animals.

## 1. Introduction

The cranial cruciate ligament (CrCL) is an important stifle component that prevents cranial tibial thrust, internal tibial rotation, and joint hyperextension [1]. Cranial cruciate ligament rupture is the most prevalent stifle orthopedic illness that requires medical care, affecting up to 4.87% of dogs [2]. However, the limited ability of CrCL regeneration and the significant force imparted to the ligament render the CrCL unable to mend when the torn ends are sutured [3]. Intra-articular graft reconstruction is frequently employed as the primary method for joint stabilization in humans [4]. In dogs, it is not often employed due to the difficulties associated with graft mechanical strength and attachment, cellular ingrowth, stifle loading following surgery, the disruption of ligament blood supply, and synovitis [5]. With the development of tissue engineering, artificial materials have emerged as viable options for ligament regeneration and repair in recent years [6]. The optimal tissue engineering graft for CrCL restoration should give initial joint stability before gradually degrading as the ligament regenerates and remodels. The scaffold is an essential component of ligament engineering because it offers mechanical support for bearing load and serves as a cell substrate for cell planting. Thus, selecting new materials and a restoration approach is vital to appropriately stabilize a CrCL-deficient stifle joint [6].

The ligament augmentation reconstruction system (LARS, Surgical Implants, and Devices, Arc-sur-Tille, France), which comprises fibers made of polyethylene terephthalate (PET), is the most widely accepted procedure for anterior cruciate ligament reconstruction in humans [7,8,9]. Although the LARS provides satisfactory clinical effects, including superior biomechanical properties, it can cause serious side effects like poor tendon–bone healing (because of the poor biocompatibility of PET materials), which limits its further clinical application [9]. CrCL repair has recently been performed using biodegradable scaffolds, including natural polymers such as collagen and silk, as well as synthetic polymers like polylactic acid (PLA), poly (lactic-co-glycolic acid) (PLGA), and polyether-urethanes (PEUUs) [6,9]. Studies confirm that these scaffolds exhibit a good histocompatibility and osteoinductivity, promoting healing between the graft and bone tunnels [6,9]. Polyurethanes are versatile synthetic polymers with numerous industrial applications. Biomer was the first polyurethane used in medicine, specifically in cardiovascular applications, due to its superior mechanical properties and favorable biocompatibility [10]. Subsequently, polyurethane was employed for CrCL repair as a synthetic graft (Artelon^®^, Artimplant AB, Västra Frölunda, Sweden) in rabbits, mini pigs, rats [11], and dogs [12]. Notably, certain medical-grade thermoplastic polyurethanes (TPUs) have demonstrated biodegradability in vivo, with degradation occurring through hydrolytic and oxidative pathways, producing non-toxic by-products such as aliphatic diols, urethane oligomers, and carbon dioxide. In vivo studies in rabbits and mini pigs have shown that PUUR, a specific medical-grade TPU, retains its mechanical integrity for up to 24 months, with degradation progressing slowly over several years via hydrolysis, supporting tissue remodeling and connective tissue ingrowth [11]. This ongoing study highlights the critical importance of selecting appropriate graft types for surgical procedures aimed at restoring stability to the canine stifle joint. It also assures the need for continuous advancements in graft materials to improve surgical outcomes.

The present cadaveric study aimed to develop a novel, biocompatible CrCL reconstruction system using 3D printing technology to fabricate an artificial ligament made from thermoplastic polyurethane (TPU). This ligament was designed with controlled properties resembling the natural CrCL for the intra-articular stabilization of a CrCL-deficient canine stifle. Additionally, the study assessed the system’s biomechanical properties and joint stability both before and after stabilization. Furthermore, a surgical plan and fixation method for joint stabilization using this system were established for future experimental and clinical applications.

## 2. Materials and Methods

The current cadaveric study was designed to evaluate the dimensions and biomechanical properties of the natural CrCL in canines. This assessment serves as a foundational model for the development of a new intra-articular reconstruction system.

### 2.1. Ethics Approval and Consent to Participate

All ethical considerations were fulfilled. Thus, ethical approval was obtained from the Institutional Animal Care and Use Committee (IACUC) of Cairo University (approval number CU II F 37 23).

### 2.2. Population

A total of 14 male, mixed-breed cadaveric dogs were included in the study. They ranged from 7 to 18 months of age, representing late juvenile to young adult stages. Prior to inclusion, each cadaver was assessed for signs of skeletal maturity through physical and radiographical examinations of growth plate closure and overall joint morphology. The dogs were euthanized at Cairo University Veterinary Teaching Hospital for reasons unrelated to the study. Within two hours post-euthanasia, the distal femoral and proximal tibial diaphyses of the cadaveric specimens were cut with an oscillating saw to separate the stifles from the limbs. Each stifle joint was then exposed following the traditional methodology of the classic Osteo Technique, which involved the removal of all soft tissues, preserving the bone segments of the stifle and the CrCL [13]. Indeed, only 21 limbs were included in the study after the exclusion of 7 limbs that were damaged during the specimen preparation process. The study meticulously evaluated the angles of CrCL inclination to the femoral and tibial axes for the 21 stifles included in the analysis. For the tensile strength and elongation measurements of the intact CrCL, only seven stifles were selected, ensuring that accurate biomechanical properties were captured. Dimension (width, thickness, and length) and volume measurements of the CrCLs were conducted on the remaining 14 stifles, which were transected at their origins and insertions. These stifles were subsequently cryogenically preserved for further study and were allocated to two distinct intraarticular reconstruction systems, with seven stifles being treated with the innovative 3D-printed TPU graft and H-button stabilization system tested in this study.

### 2.3. Biomechanical Testing of Natural, Intact CrCL

The stifle joint specimens (*n* = 7) were not frozen and were ready for mechanical testing following euthanasia. All soft tissue was meticulously removed, leaving only the natural CrCL. The femoral and tibial bone parts were securely attached to electronic universal testing equipment. (AGS-X, Shimadzu, Kyoto, Japan). The ligament was stretched at a 6 mm/min rate until it burst. The tensile strength and elongation at break were then determined [14].

### 2.4. Components of the Intraarticular CrCL Reconstruction System

The innovative intra-articular CrCL reconstruction system consisted of an artificial biodegradable graft (3D-TPU), cerclage wire connectors, and H-button fixators.

### 2.5. Preparation of the 3D-Printed TPU Graft

**Figure 1:**
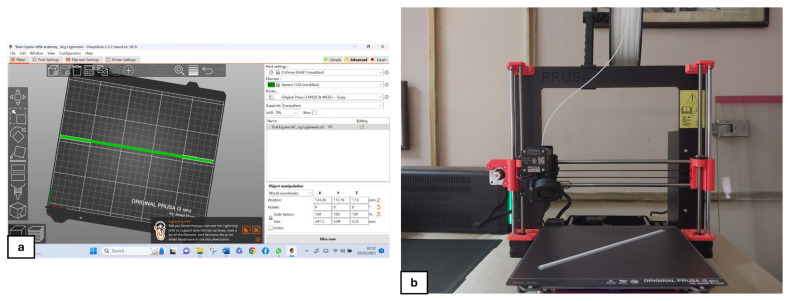
Processing and printing of the TPU graft. (**a**) Processing of the graft model using PrusaSlicer software and (**b**) printing of the graft using the Original Prusa i3 MK3S+ 3D printer.

A natural, intact CrCL was scanned using the “EINSCAN PRO 2X/2X PLUS” portable 3D scanner from SHINING 3D Tech Co., Ltd. (Hangzhou, China). The volume of the 3D-TPU graft was designed to resemble the natural CrCL using Blender version 4.2.3 LTS. The digital files were processed and refined using PrusaSlicer 2.5.2 software (Figure 1a) (Josef Prusa, Prague, Czech Republic), with the parameters outlined in Table 1. This step ensured image correction and model optimization for accurate 3D printing. Autodesk Meshmixer© (Autodesk Inc., San Rafael, CA, USA) was utilized to reduce image noise. The optimized images obtained after scanning and editing were then printed using a Prusa i3 MK3S+ 3D printer^®^ (Josef Prusa, Prague, Czech Republic) (Figure 1b) [15,16,17,18]. Thermoplastic polyurethane filament (e sunTPU-95A) was utilized during the printing process, with a temperature setting of 200 °C. Different graft samples were produced with varying internal fill percentages of 5%, 10%, and 15%, which were subsequently subjected to mechanical testing. This process enabled the identification of the infill percentage that provided the best balance of tensile strength and elasticity. The selected optimal infill percentage was then utilized for the grafts used in the reconstruction of the cadaveric specimens.

### 2.6. Biomechanical Testing of the Raw 3D-Printed TPU Graft

Mechanical testing of the grafts (*n* = 7) was conducted using universal Galdabini apparatus (Giovanni XXIII, 183-21010 Cardano al Campo—Varese, Italy), capable of applying a tensile strength ranging from 2500 to 50,000 N. During the testing, the grafts were elongated at a rate of 70 mm per minute, and both the tensile strength and elongation at break were meticulously recorded [14].

### 2.7. Micromorphology of the 3D-Printed TPU Graft

Using a sputter coater (S150A; Edwards, Crawley, United Kingdom), the TPU graft was coated with gold and then scanned using a field emission scanning electron microscope (Quanta FEG250; Thermo Fisher Scientific; Waltham, MA, the US) at a 1 kV acceleration voltage.

### 2.8. Preparation of the H-Button Fixator

A customized button plate was made from stainless steel 316L utilizing a laser cutting machine after being designed in AutoCAD 2022 software (Blue Elephant CNC, China).

### 2.9. Stifle Specimen Stabilization Procedure

**Figure 2:**
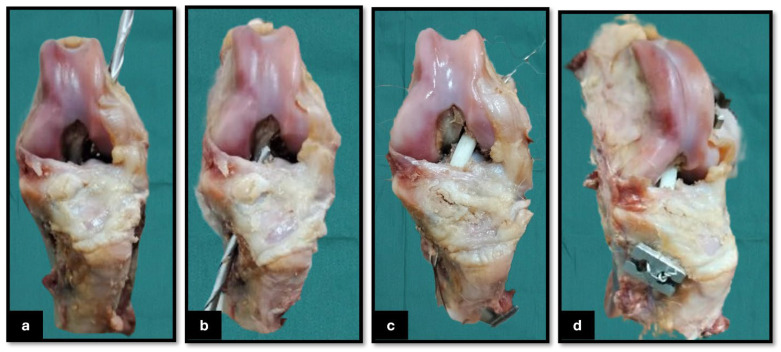
Surgical procedure for cranial cruciate ligament (CrCL) reconstruction in a cadaveric canine limb. (**a**) Drilling of the femoral tunnel from the lateral femoral cortex toward the intra-articular origin of the CrCL; (**b**) drilling of the tibial tunnel from the medial tibial cortex toward the CrCL insertion site within the joint; (**c**) passage of the 3D-printed TPU graft connected with the cerclage wire through both bone tunnels, with the graft positioned within the intra-articular space before being fixed on the femoral and tibial H-button plates; and (**d**) craniomedial view of the final reconstructed stifle joint, demonstrating securing the intra-articular graft on the H-button plates.

The seven stifle specimens underwent a defrosting period of 72 h at 4 °C. Following this, a 5 mm femoral tunnel was meticulously drilled from the lateral side of the lateral femoral condyle, ensuring that it was aligned through the center of the CrCL origin footprint. This was accomplished using a 5 mm cannulated drill bit in conjunction with a 6 mm drill guide provided by Arthrex, Naples, FL (Figure 2a). Following this, a corresponding 5 mm tibial tunnel was created from the medial tibial cortex, also targeting the center of the CrCL insertion footprint (Arthrex, Naples, FL) (Figure 2b). The lengths of both the femoral and tibial tunnels were subsequently measured to approximately calculate the required length of the graft for proper reconstruction. The TPU 3D-printed graft was fixed using a 5 mm cerclage wire (Figure 2c) as a connector incorporated into the two ends of the graft and then hinged on two H-buttons to be fixed against the lateral femoral condyle and medial cortex of the tibia (Arthrex, Naples, FL) (Figure 2d). The stabilization procedure was conducted on the selected stifle joints in a state of extreme flexion. After the reconstruction, the effectiveness of stabilization was assessed by conducting the cranial drawer test to confirm joint stability.

### 2.10. Biomechanical Testing of the Intraarticular CrCL Reconstructed Stifles

Biomechanical testing of the stabilized joint specimens (*n* = 7) was performed under uniaxial tensile cyclic stress. All soft tissues were carefully excised, leaving only the CrCL reconstruction system in place. The distal femoral and proximal tibial bone segments were firmly secured in an electronic universal materials testing system (AGS-X, Shimadzu, Kyoto, Japan). The experiment utilized an elongation rate of 6 mm/min. The tensile strength and elongation at which the graft failed (either by bursting or being pulled out from the bone tunnels) were measured [14].

### 2.11. Statistical Analysis

Data analysis was performed using commercial statistical software (GraphPad Prism Version 10.3.0 (507), La Jolla, CA, USA). Data were evaluated for normality using the Kolmogorov–Smirnov test and were determined to be normally distributed. Bartlett’s test was performed to evaluate homogeneity of variance. Means (±SDs), ranges, and 95% confidence intervals (CIs) were calculated for all datasets. The non-parametric ANOVA test (Kruskal–Wallis’s test) and Dunn’s multiple comparisons test were utilized to compare the tensile strength (N) and elongation (%) of the intact CrCL against the raw TPU and the TPU following joint stabilization (implanted TPU). The Pearson correlation coefficient was employed to analyze the relationship between the volume of the CrCL and both the age and body weight of the enrolled dogs. Multivariant Linear regression analysis was also performed to determine the influence of body weight and age on CrCL volume. A significance level of *p* < 0.05 was established.

## 3. Results

### 3.1. Population

**Figure 3:**
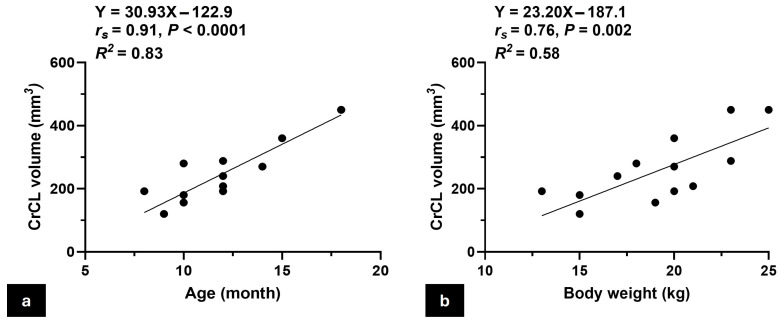
Scatter plots show the correlation between CrCL volume and animal characteristics. (**a**) A scatter plot illustrates the positive correlation between the CrCL volume and the age of the animal, indicating an increase in CrCL volume with age; (**b**) scatter plot showing the positive correlation between CrCL volume and body weight, demonstrating that larger animals tend to have greater CrCL volumes. **Note:** The number of visible data points in Figure 3a,b appears lower than the total number of animals (*n* = 14) due to overlapping values; some dogs shared identical age or body weight, resulting in superimposed points on the graph.

The study involved fourteen adult male mixed-breed cadaveric dogs with an average age of 12.1 ± 3.2 months and an average weight of 18.9 ± 3.5 kg, contributing to a total of 21 stifle joints examined. Morphological measurements of the CrCLs, including length, width, thickness, and estimated volume, were obtained from both the right and left stifles of 14 dogs. Detailed demographic data and ligament morphometrics are summarized in Table 2. Initial correlation analysis revealed a significantly strong positive correlation between CrCL volume and both age and body weight, with Spearman’s rank correlation coefficients (rs) of 0.91 (*p* < 0.0001) for age and 0.76 (*p* = 0.002) for body weight (Figure 3). To control for potential confounding between these variables, a multivariate linear regression analysis was conducted with CrCL volume as the dependent variable and age and body weight as independent predictors. The model was statistically significant (F(2,11) = 26.79, *p* < 0.001), with an R^2^ of 0.83, indicating that approximately 83% of the variability in CrCL volume could be explained by the combined effects of age and body weight. Age was found to be a significant predictor (β = 31.12, *p* = 0.002), whereas body weight was not (β = −0.21, *p* = 0.98).

### 3.2. Dimensions, Inclination, and Biomechanics of Intact CrCL

The means (±SD), ranges, and 95% CIs for the length, width, thickness, and volume of the intact CrCL, as well as the CrCL inclination angles relative to the corresponding distal femoral and proximal tibial axes, along with the femoral and tibial tunnel measurements, are presented in Table 3. The tensile strength of the CrCL was 518.4 ± 97.5 N, and its elongation at break was 49.3 ± 5.3%.

### 3.3. Intra-Articular CrCL Reconstruction System

**Figure 4:**
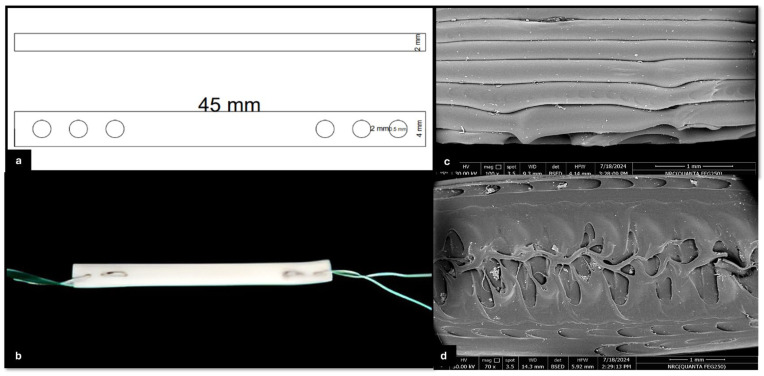
Structural and morphological characterization of the 3D-printed TPU graft. (**a**) Schematic diagram showing the top and side views of the TPU graft design. The graft has a total length of 45 mm, a thickness of 2 mm, and includes multiple holes with diameters of 2 mm and 5 mm for cerclage wire passage and fixation; (**b**) 3D-printed TPU graft connected at both ends with cerclage wire; (**c**) high-magnification electron microscopy images showing the surface morphology of the TPU fibers and inter-fiber architecture; and (**d**) SEM image showing the internal microstructure of the 3D-printed TPU graft, highlighting the interconnected porosity and inner architecture. A 1 mm scale bar is included in the lower right corner.

**Figure 5:**
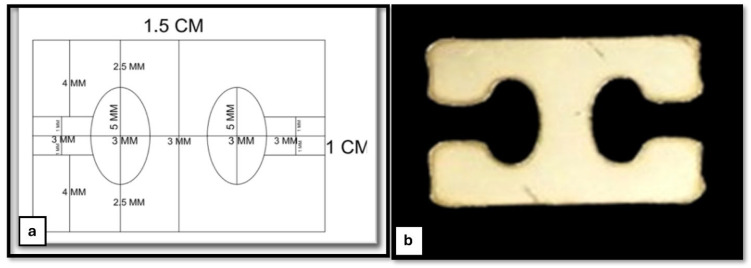
Stainless-steel H-button plate. (**a**) Schematic diagram of the H-button plate, illustrating its design and dimensions and (**b**) the actual stainless-steel button plate, showing its structural features.

This reconstruction system comprises a 3D-printed TPU graft, cerclage wire (connector), and two H-buttons (Figure 4b). The 3D-printed TPU graft has a thickness of 2 mm and a width of 4 mm, with an infill density of 15%. Each end of the TPU graft contains three holes, designed for secure fixation using a 5 mm cerclage wire on both sides to be hinged on the two H-buttons during joint stabilization (Figure 4a). The H-button is 15 mm long, 10 mm wide, and 2 mm thick. It contains two open eyes of 5 mm long and 3 mm wide each. There is a 3 mm long, 2 mm wide slit related to each eye. The septum between the two eyes is 3 mm wide (Figure 5).

### 3.4. Micromorphology and Biomechanics of Raw and Implanted 3D-Printed TPU Graft

**Figure 6:**
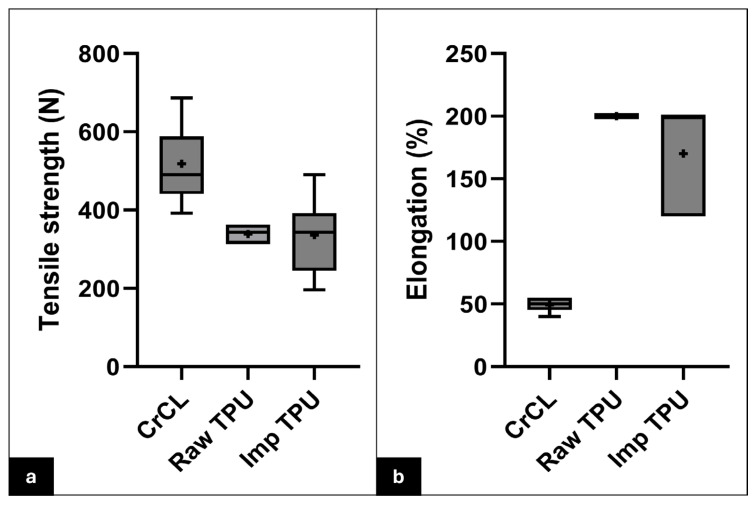
Whisker plots comparing the mechanical properties of the natural CrCL, raw TPU, and implanted TPU. Box-and-whisker plots illustrate the distribution of tensile strength (**a**) and elongation at break (**b**) among the three groups: intact CrCL, raw TPU, and implanted TPU. The boxes represent the interquartile range (IQR), with the horizontal line inside each box indicating the median. Whiskers extend to the minimum and maximum values within 1.5 times the IQR. The “+” symbol represents the mean. Note: The raw TPU group in panel (**b**) exhibits minimal variation in elongation at break, which results in a near-zero IQR. Sample size for each group: CrCL (*n* = 7), raw TPU (*n* = 7), implanted TPU (*n* = 7).

The TPU filaments exhibited a relatively smooth surface of elongated polymer strands, showing layer-by-layer deposition. A few micro-voids or air pockets were found between the layers due to low infill percentages (Figure 4c,d). The tensile strength of the raw 3D-printed TPU was 339.0 ± 24.6 N. The elongation at break was >200%. The tensile strength and elongation at break of the implanted 3D-printed TPU for the reconstructed cadaveric stifle specimens were 336.2 ± 96.4 N and 170 ± 38.73%, respectively. There was a significant variation in tensile strength (rs = 0.56, *p* = 0.0006) and elongation at break (rs = 0.90, *p* < 0.0001) among the following three groups: the intact CrCL, raw TPU, and implanted TPU (Figure 6). Regarding tensile strength, Dunn’s multiple comparisons test indicated that the intact CrCL group differed significantly from both the raw TPU group (*p* = 0.005) and the implanted TPU group (*p* = 0.014). However, no significant difference was found between the raw TPU and implanted TPU groups (*p* > 0.999). For elongation, the intact CrCL group showed significantly lower elongation compared to both the raw TPU group (*p* = 0.0003) and the implanted TPU group (*p* = 0.0099). The difference between the raw and implanted TPU groups was not statistically significant (*p* = 0.9824).

## 4. Discussion

CrCL rupture is the most prevalent cause of stifle joint lameness in dogs, leading to instability and inflammation and contributing to osteoarthritis over time [9]. In the 1950s, an intra-articular method employing an autograft was used to repair a torn CrCL ligament in dogs [19]; however, the procedure was not extensively adopted because of donor site morbidity and graft necrosis [12]. Graft selection is fundamental to CrCL rehabilitation [9]. The ligament augmentation reconstruction system (LARS, Surgical Implants, and Devices, Arc-sur-Tille, France) has emerged as the most widely used synthetic graft made of PET fibers (non-degradable fibers) in recent years [7,8,9]. However, it may result in negative consequences such as synovitis, loosening, and poor tendon–bone repair [20,21]. While these limitations have been highlighted in previous studies, the current investigation was limited to cadaveric specimens and did not involve in vivo biological assessment. Additionally, PET grafts were not included as a comparative control under the same biomechanical testing conditions. Therefore, future studies incorporating PET controls and in vivo models are necessary to validate the clinical advantages of PU over PET. ACL/CrCL restoration recently proved the potential of biodegradable scaffolds to promote the healing process between the utilized graft and bone tunnels [9]. Synthetic biodegradable polyesters such as polylactic acid (PLA) and polyglycolic acid provide an easy supply and a reliable, repeatable procedure.

Artelon^®^, a biocompatible and biodegradable polyurethane graft, has been used to repair CrCL in rabbits, mini pigs [11], and dogs [12]. These investigations demonstrated the effective restoration of stifle function, with histological assessment confirming biocompatibility and tissue ingrowth between fibers [11,12]. Recently, 3D printing technology was utilized for reconstructing the ACL [22,23,24,25,26,27,28]. The present study developed a 3D-printed TPU graft manufactured from modified TPU 95A filament (e sunTPU-95A, USA), a semi-flexible, chemically resistant filament with good layer bonding and an acceptable tensile strength value (39.0 MPa). Additionally, it is quicker and easier to print than other TPU fibers. The limited tensile strength of the 3D-printed TPU graft before (339.0 ± 24.6 N) and after reconstruction (336.2 ± 96.4 N), comparable to the natural CrCL (518.4 ± 97.5 N), may refer to the percentage of infill of the graft (15%) used in the current study. However, increasing the infill percentage would enhance the tensile strength, but would also reduce the elasticity of the final implant. Despite the relatively lower tensile strength of the produced graft, it exhibited elongation without rupture, even after reaching its tensile strength limit.

Graft fixation aims to stabilize the graft within the bone tunnel until it is incorporated into the bone [29]. Several methods for graft fixation have been reported; direct methods include absorbable and non-absorbable interference screws, cross pins, staples, washers, and hardware-free press-fit fixation, while indirect methods include permanent or adjustable suspensory cortical button attachment [29]. Suspensory (button) fixation provides advantages such as technique simplicity, a larger graft-to-bone contact area for improved integration, and a higher fixation strength and stiffness [29,30,31]. Fixed loop suspension has better biomechanical effects than adjustable loop suspension [32]. In the current study, the H-button plate was developed with two open eyes that made it simple to roll up the graft. The eyes’ diameters were also appropriate for the graft volume in the case of doubling the graft within the joint. The cerclage wire was used to secure the scaffold in the fixation method. Although its use in scaffold fixation within the stifle joint has not been reported previously, it did not result in any tears or damage to the graft at the insertion sites. Additionally, it can provide multi-point fixation, offering both biomechanical stability and tissue support during healing. However, this biomechanical setup does not allow for the assessment of biological or long-term responses. The potential risks associated with intra-articular cerclage wire, including synovial irritation, chronic inflammation, mechanical wear, and wire migration, must be carefully considered. Future in vivo studies incorporating imaging and histological evaluation will be essential to investigate the long-term biocompatibility and mechanical safety of this fixation method and to determine its clinical applicability.

The present study comprehensively analyzed the dimensions (length, width, and thickness), volume, inclination angles relative to the femoral and tibial axes, tensile strength, and elongation of the CrCL. These findings provide a strong foundation for the development of an intra-articular reconstruction system and offer valuable insights for future experimental and clinical research. The average CrCL length recorded in this study was 13.14 mm, which aligns well with the previous literature, where the reported lengths for paired stifle joints range from 7.88 to 23.16 mm [33]. However, a notable gap in prior veterinary studies is the lack of explicit width measurements. This omission may stem from the inherent variability in CrCL width at different anatomical locations, making accurate assessments challenging and preventing the establishment of a standardized value. To address this limitation, the present study standardized width measurements at the midpoint of the CrCL to ensure consistency and improve comparability across future research. This study also emphasized the inclination angle of the CrCL relative to the femoral and tibial axes, underscoring the importance of precise surgical techniques, particularly in the creation of bone tunnels. The findings further clarified the variation in tunnel lengths between the tibia and femur, highlighting the need for meticulous planning during reconstruction. A strong positive correlation was observed between CrCL volume and age (r = 0.90, *p* < 0.0001), consistent with previous studies [34]. The strong positive correlation observed between CrCL volume and age may partially reflect natural growth-related increases in ligament dimensions as dogs progress from the late juvenile period to early adulthood. Although a few specimens were 7–8 months old, all dogs were determined to be skeletally mature or approaching full maturity. Thus, the observed variability in CrCL volume across the age range likely represents a continuum of anatomical development rather than immature outliers.

In addition, we acknowledge that intrinsic age-related tissue remodeling could also influence CrCL structure, even within this relatively narrow age range. Therefore, the correlation should be interpreted as reflecting both physiological growth and early remodeling processes. Additionally, the correlation between CrCL volume and body weight (r = 0.72, *p* = 0.004) aligns with recent findings by Brockmeyer et al. (2023), reinforcing the relationship between body weight and CrCL volume in canine patients. These correlations highlight the significance of age and body weight in assessing CrCL health and surgical considerations, emphasizing their role in optimizing treatment strategies for canine patients [35].

This study acknowledges the absence of female subjects as a limitation, recognizing the importance of sex differences in future research on CrCL reconstruction. While the current design was optimized for medium-sized dogs, anatomical variability across breeds, particularly in smaller dogs, may impose technical limitations due to reduced joint space or bone tunnel size. Additionally, the focus on a single breed (all specimens were from mixed-breed dogs) presents another constraint, as the designed intra-articular reconstruction system may not be universally applicable to other breeds. These factors highlight the need for future investigations to explore breed-specific adaptations and evaluate the feasibility of the reconstruction system across a broader range of canine sizes and morphologies. Further experimental and clinical studies should focus on validating the intra-articular stabilization system and procedure, assessing clinical and biomechanical outcomes, and monitoring implant–bone integration, graft remodeling, ligamentization, and joint adaptation to optimize its effectiveness. The lack of quantitative surface roughness measurements (e.g., Ra and Rq values) and detailed pore characterization is considered another limitation. Although scanning electron microscopy (SEM) revealed surface micro-voids, advanced analyses using profilometry, atomic force microscopy (AFM), or micro-computed tomography (micro-CT) were not available during the study period. These techniques would provide a more precise evaluation of surface topology and internal porosity, which are critical factors influencing scaffold performance, mechanical fatigue, and biological response. Future studies should incorporate such analyses to optimize surface properties and assess the effects of potential post-processing strategies, including polishing, chemical smoothing, and bioactive coatings. This study did not evaluate the direct relationship between individual CrCL dimensions (e.g., length, width, and thickness) and ligament strength. While these anatomical parameters are known to influence mechanical behavior, the present work focused on the correlation between CrCL volume and systemic factors such as age and body weight. Future studies are warranted to explore the biomechanical implications of individual dimensional variations. Additionally, this study was conducted on a relatively small sample size, with only seven specimens per group. This number was selected based on prior pilot studies and in accordance with ethical guidelines aimed at reducing the use of animal cadavers. However, we acknowledge that this limited number may not fully represent the anatomical variability among different individuals, particularly in parameters such as femoral inclination angle or cruciate ligament morphology. Future studies with larger sample sizes are warranted to validate these findings and better understand the influence of anatomical diversity on biomechanical and morphological outcomes.

## 5. Conclusions

In conclusion, this study developed an innovative intra-articular CrCL reconstruction system for dogs with CrCL-deficient stifles. The system consisted of a 3D-printed TPU graft, cerclage wire (connector), and two H-buttons as fixators. While the design showed promise based on morphological and mechanical assessments, further experimental research is needed to evaluate its effectiveness in implant–bone healing, graft remodeling, and ligamentization, as well as to assess joint stability and clinical and biomechanical outcomes in reconstructed stifle joints.

## 6. Patents

A patent application has been filed for the novel intra-articular cranial cruciate ligament reconstruction system described in this study. The application was submitted under the Egyptian Patent Office with the request number EG/P/2025/545.

## Figures and Tables

**Table 1:** Three-dimensional printing parameters for the TPU graft fabrication.

Software Parameters		Printer Parameters	
**Support**	NO	**Temp**	200 °C
**Infill**	15%	**Time**	25 MIN
**The material used**	TPU	**Cost**	300 EGP
**Nozzle**	0.15 mm to increase accuracy	**Nozzle**	0.4 mm
		**Type of bed**	Texture sheet

**Table 2:** Demographic and morphological data of canine specimens (right and left CrCLs).

Dog ID	Age (mo)	Weight (kg)	Limb Side	CrCL Length (mm)	Width (mm)	Thickness (mm)	Volume (mm^3^)
1	18	25	Right	15	10	3	450
2	14	20	Right	15	9	2	270
			Left	14	8	2	224
3	18	23	Right	20	10	3	600
			Left	19	9	3	513
4	10	19	Right	13	6	2	156
5	8	13	Right	12	8	2	192
6	10	15	Left	15	6	2	180
7	12	20	Right	12	8	2	192
			Left	11	7	2	154
8	15	20	Right	15	8	3	360
			Left	15	8	2.5	300
9	12	23	Right	12	8	3	288
			Left	12	7.5	3	270
10	9	15	Right	10	6	2	120
			Left	10	6	2	120
11	12	21	Right	13	8	2	208
12	10	18	Left	20	7	2	280
			Left	19	7	2	266
13	12	17	Right	15	8	2	240
14	12	14	Right	15	8	3	360

**Table 3:** Morphological characteristics of the cranial cruciate ligament (CrCL) based on cadaveric stifle specimens collected from 14 dogs. Descriptive statistics of the CrCL and tunnel measurements, including width, thickness, length, volume, inclination angles, and tunnel lengths. Data are presented as mean ± standard deviation (SD), range, and 95% confidence intervals (CIs).

Parameter	Number of Values	Mean ± SD	Range	95% CI (Lower—Upper)
**CrCL Width (mm)**	14	7.5 ± 1.3	6–10	6.92–8.128
**CrCL Thickness (mm)**	14	2.3 ± 0.5	2–3	2.075–2.496
**CrCL Length (mm)**	14	13.4 ± 2.9	10–20	12.04–14.72
**CrCL Volume (mm^3^)**	14	250.4 ± 107.8	120–450	188.2–312.6
**CrCL Femoral Inclination Angle (°)**	21	35.7 ± 5.3	25–45	33.3–38.13
**CrCL Tibial Inclination Angle (°)**	21	33.8 ± 6.1	25–45	31.03–36.59
**Femoral Tunnel Length (FTL, mm)**	14	20.7 ± 1.8	20–25	19.67–21.76
**Tibial Tunnel Length (TTL, mm)**	14	24.7 ± 2.1	20–30	23.53–25.9

## Data Availability

All data generated or analyzed during this study are included in this published article.

## Abbreviations

The following abbreviations are used in this manuscript:

ACL	Anterior cruciate ligament
CrCL	Cranial cruciate ligament
IACUC	Institutional Animal Care and Use Committee
LARS	Ligament augmentation reconstruction system
PET	Polyethylene terephthalate
PEUUs	Polyether-urethanes
PLA	Polylactic acid
PLGA	lactic-co-glycolic acid
TPU	Thermoplastic polyurethane
